# Conditional Deletion of Smad1 Ameliorates Glomerular Injury in Progressive Glomerulonephritis

**DOI:** 10.1038/srep31216

**Published:** 2016-08-05

**Authors:** Makoto Araki, Takeshi Matsubara, Hideharu Abe, Kazuo Torikoshi, Akira Mima, Noriyuki Iehara, Atsushi Fukatsu, Toru Kita, Hidenori Arai, Toshio Doi

**Affiliations:** 1 Department of Nephrology, Kyoto University, Kyoto, Japan; 2 Department of Nephrology, Institute of Biomedical Sciences, Tokushima University Graduate School, Tokushima, Japan; 3 Kobe City Medical Center General Hospital, Kobe, Japan; 4 National Center for Geriatrics and Gerontology, Obu, Japan

## Abstract

Matrix expansion and cell proliferation are concomitantly observed in various glomerular injuries. However, the molecular mechanisms responsible for these changes have not been fully elucidated. We have reported that Smad1 is a key signalling molecule that regulates the transcription of type IV collagen (Col4) in mesangial matrix expansion and is thereby involved in glomerular injury in an acute model of glomerulonephritis. In this study, we addressed the role of Smad1 signalling in accelerated nephrotoxic nephritis (NTN), a model of progressive glomerulonephritis, using conditional deletion of *Smad1* in *Rosa26CreERT2* mice (*Smad1*-CKO). Mesangial matrix expansion in the *Smad1*-CKO mice with NTN was significantly inhibited compared with that in wild type mice with NTN, which was consistent with the decrease in Col4 expression level. On the other hand, STAT3 activation and cell proliferation were not influenced by *Smad1* deletion in the NTN model. Therefore, we investigated another factor that activates cell proliferation in the absence of Smad1. Id2 induced VEGF secretion and subsequent STAT3 activation, independently of Smad1 expression in mouse mesangial cells. Here we show that Smad1 plays an important role in the development of glomerular injury without affecting cell proliferation, in progressive glomerulonephritis.

The global burden of end-stage renal disease is a significant public health threat. Therefore, it is urgent that researchers elucidate the mechanisms of kidney disease and develop adaptive treatment strategies. Glomerulonephritis (GN) is a common cause of end-stage renal disease and glomerulosclerosis is defined as the segmental or global collapse or closure of capillary loops with associated extracellular matrix (ECM) overproduction in the mesangial area. Excessive proliferation of cells and subsequent overproduction of ECM contribute significantly to the pathogenesis of GN and glomerulosclerosis. Mesangial matrix expansion is characterized by increased deposition of ECM, such as type IV collagen (Col4), laminin, type I and III collagens, heparan sulphate proteoglycan, and fibronectin^1^,^2^,^3^. Among these factors, Col4 is the most important component of the mesangial matrix and is distributed in all layers of the basement membrane, forming its structural frame.

We have previously reported that Smad1 transcriptionally regulates the expression of Col4 under diabetic conditions *in vitro* and *in vivo*^4^. Moreover, we have shown that administration of anti-PDGFβ-receptor (anti-PDGFβR) antibody (APB5) to the mesangial-proliferative model of GN in Thy1 GN rats attenuates glomerular cell proliferation and glomerulosclerosis and Smad1 activation. However, the Thy1 GN model is self-limiting and spontaneously reversible. In contrast, most serious glomerular diseases develop more progressively and result in kidney failure. Therefore, it is necessary to investigate whether blockade of PDGFβR can exert a similar effect in progressive forms of GN. In this study, we analysed a well-established model of severely progressive GN, induced nephrotoxic nephritis (NTN), using conditional *Smad1* knockout (*Smad1*-CKO) mice.

It is well known that platelet-derived growth factor BB (PDGF-BB) plays a central role in GN^5^,^6^,^7^, and pharmacological administration of PDGF-BB causes mesangial cell (MC) proliferation and ECM accumulation^7^. We have also shown that PDGF-BB phosphorylates Smad1 and induces the expression of Col4 via signal transducer and activator of transcription 3 (STAT3) in cultured MCs^8^. However, the functional role of Smad1 and its relationship with the PDGF-BB–STAT3 signalling pathway in the development of GN *in vivo* remains to be elucidated. Moreover, it remains unclear which molecules determine the progression or remission of GN.

To address these issues, we created and validated *Smad1*-CKO mice and used them to examine the role of Smad1 in murine NTN. In addition, we investigated the relationship between Smad1 activation and the PDGF-BB signalling pathway. We confirmed that Smad1 is essential for progressive glomerular injury in this model. Furthermore, we found that novel molecular mechanisms underlie the Smad1-independent signalling pathway and activate cell proliferation and STAT3.

## Results

### Morphological glomerular changes and activation of PDGF signalling pathway in glomeruli in NTN

**Figure 1:**
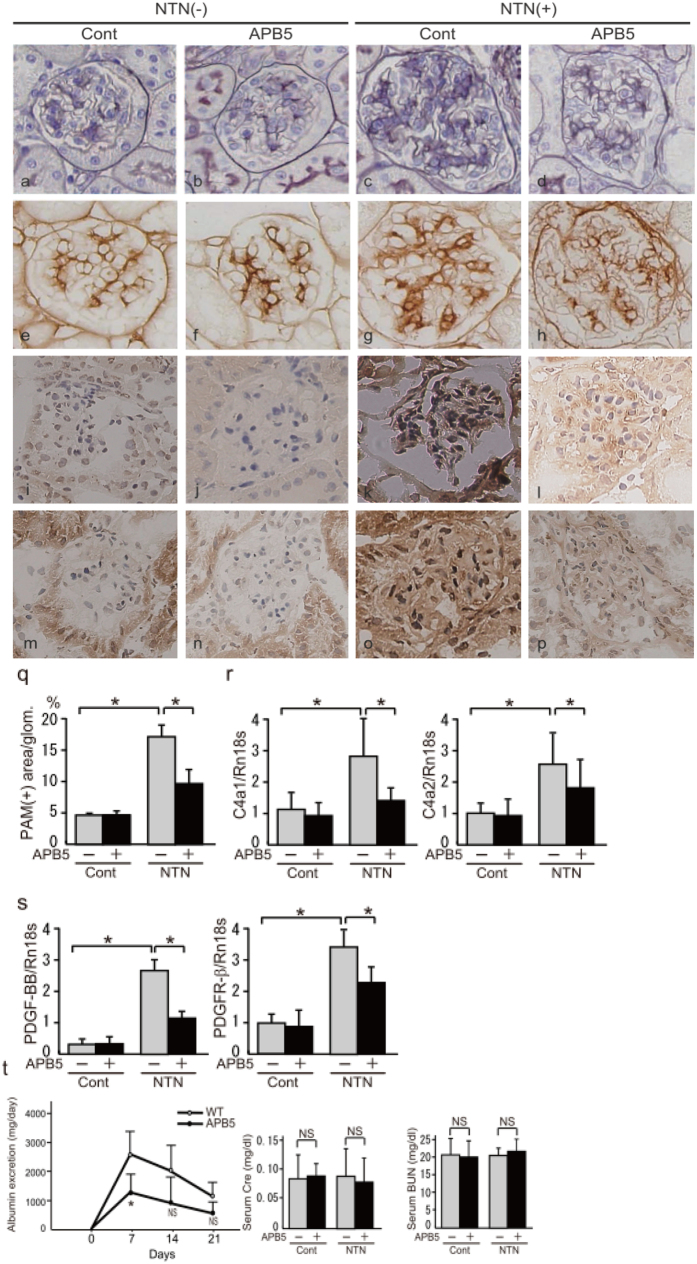
Morphological changes and effects of PDGFβR inhibition in NTN. The microscopic lesions in NTN mice appeared as the diffuse proliferation of mesangial matrix and expansion of the mesangial area. Immunohistochemical staining with anti-Col4 antibodies revealed the overexpression of Col4 in the expanded mesangial area of glomeruli from kidneys of NTN mice. NTN mics also showed significantly enhanced expression of PDGF-BB and PDGFβR in the glomeruli. Representative microscopic appearance of the glomerulus is shown. (**a**–**d**) PAM. (**e**–**h**) Col 4. (**i**–**l**) PDGF-BB. (**m**–**p**) PDGFβR. Original magnification for all panels was ×400. (**q**) PAM staining-positive area of the glomeruli. We collected at least 20 glomeruli in each sample. The expression levels of Col4 (Col4a1 and Col4a2) (**r**) and PDGF signals (**s**) in the glomeruli 21 d after the initial NTS injection were analysed by qPCR and normalized to the expression of Rn18s. The values are expressed as the means ± SD. *P < 0.05. (**t**) Time course of changes in urinary albumin excretion in the two groups – control IgG- (○) and APB5- (●) treated mice, and serum Cre and BUN levels at day 21. Statistical analysis for comparison was performed using unpaired two-tailed Student’s *t*-test. NS, not significant, *P < 0.05 (n = 5 mice/group).

NTN is used as a model of acute and progressive renal injury^9^,^10^. Soon after Nephrotoxic serum (NTS) injection, mice rapidly develop severe glomerulonephritis with hypertrophy, and glomeruli are replaced by fibrotic tissue. We have previously reported that Thy1 rat model exhibit remission of both proliferative changes and ECM expansion simultaneously^8^. Therefore, our aim in this study was to gain some clues for molecular mechanisms underlying the difference between reversible and irreversible proliferative changes in glomerulonephritis. For this study, we sacrificed the mice 21 days after the injection, corresponding to the change from glomerular hypertrophy to glomerulosclerosis^11^ (Fig. 1a–d,q). Microscopic analysis of NTN showed diffuse proliferation of the mesangial matrix and an expansion of the mesangial area (Fig. 1c). In addition, immunohistochemical examination demonstrated the overexpression of Col4 in the expanded mesangial area of glomeruli (Fig. 1g). NTN also exhibited significantly enhanced expression of PDGF-BB and PDGFβR in the glomeruli (Fig. 1k,o). The APB5 monoclonal antibody specifically binds to PDGFβR and blocks PDGF signalling as described previously^12^,^13^. We treated NTN mice with APB5 to block PDGFβR-mediated signalling because PDGF-BB is involved in ECM expansion and proliferative changes. As expected, NTN mouse glomeruli exhibited glomerulosclerosis with marked ECM expansion and cell proliferation. APB5 treatment significantly inhibited the glomerular hypertrophy (Fig. 1c,d,q). As shown in Fig. 1a, this glomerular hypertrophy was not affected by the control antibody treatment. We isolated glomeruli using laser-captured microdissection (LCM) techniques, extracted mRNA, and analysed gene expression in the glomeruli. Expression of Col4 in the glomeruli showed consistent results with quantitative RT-PCR (qPCR) analysis (Fig. 1r). Treatment with APB5 significantly suppressed the overexpression of both PDGF-BB and PDGFβR (Fig. 1l,p,s). Correlation between the expressions of phospho-Smad1 (pSmad1) and Col4 was expected based on our previous study. Unlike these inhibitory effect of APB5, the significant amelioration of renal function was observed in albumin excretion at day 7 alone, implying that compensatory response may be induced in this model (Fig. 1t). We conducted experiments using the *Smad1*-CKO mice to prove the direct relationship between Smad1 and Col4 *in vivo*.

### Expression of Smad1 and pSmad1 in glomeruli in NTN

**Figure 2:**
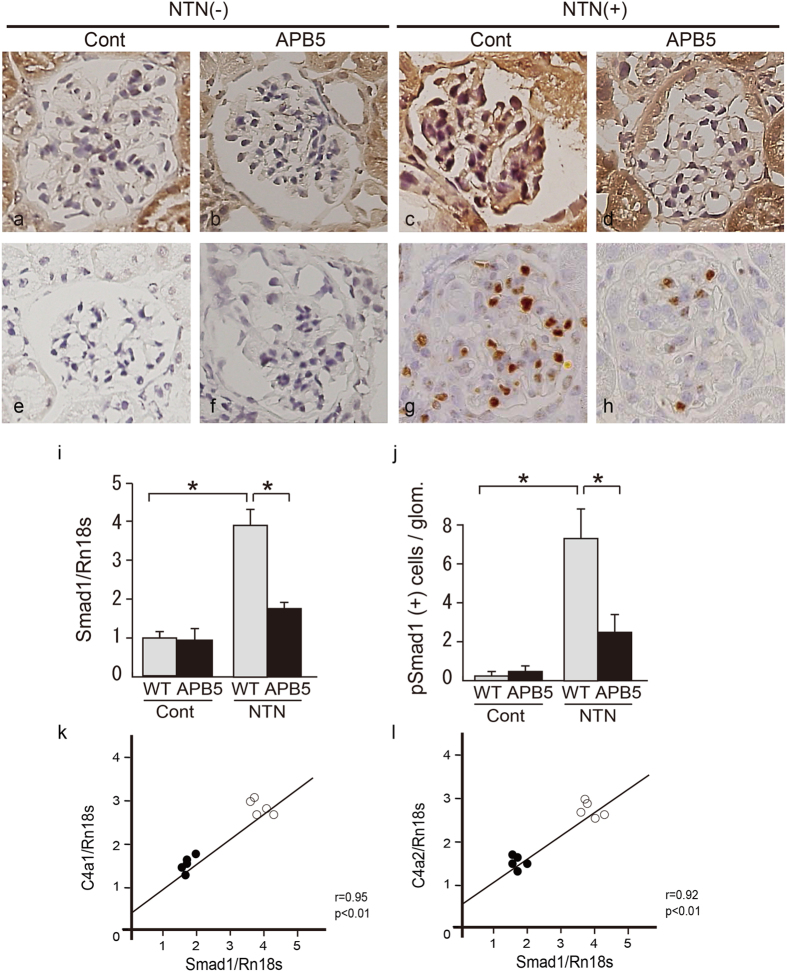
Effects of PDGFβR inhibition on the induction and activation of Smad1 signal in NTN. Immunohistochemical staining of Smad1 and pSmad1 in glomeruli in NTN. NTN also showed significantly enhanced expression of Smad1 and pSmad1 in the glomeruli. APB5 treatment led to a significant decrease in expressions of these proteins. (**a**–**d**) Smad1. (**e**–**h**) pSmad1. (**i**) The expression levels of Smad1 in the glomeruli 21 d after the initial NTS injection were analysed by qPCR and normalized to the expression of Rn18s. The values are expressed as the mean ± SD. *P < 0.05. (**j**) The number of cells positive for pSmad1. Fifty glomeruli were analysed for each sample. The values are expressed as the mean ± SD. *P < 0.05. Regression analysis comparing the changes in pSmad1 levels with changes in Col4a1 (**k**) and Col4a2 (**l**) expression mRNA levels in NTN model mice treated with control IgG (○) and APB5 (●).

We examined Smad1 signal activation in NTN mice by immunohistochemical analysis. Smad1 and pSmad1 were barely detectable in control glomeruli and glomeruli treated with APB5 alone (Fig. 2a,b,e,f). Although Smad1 was extensively induced and activated in the glomeruli at day 21 in NTN mice (Fig. 2c,g), the activation of Smad1 signallings was inhibited by APB5 treatment (Fig. 2d,h). We then performed a qPCR using mRNA from isolated glomeruli and found that both the expression of Smad1 and the number of pSmad1–positive cells were suppressed by APB5 treatment in proportion to the reduction in Col4 (Fig. 2i,j). The mRNA expression levels of Smad1 was significant correlated with those of Col4 in NTN treatment both with and without APB5 administration (Fig. 2k,l).

### Generation of *Smad1* conditional knockout mice

**Figure 3:**
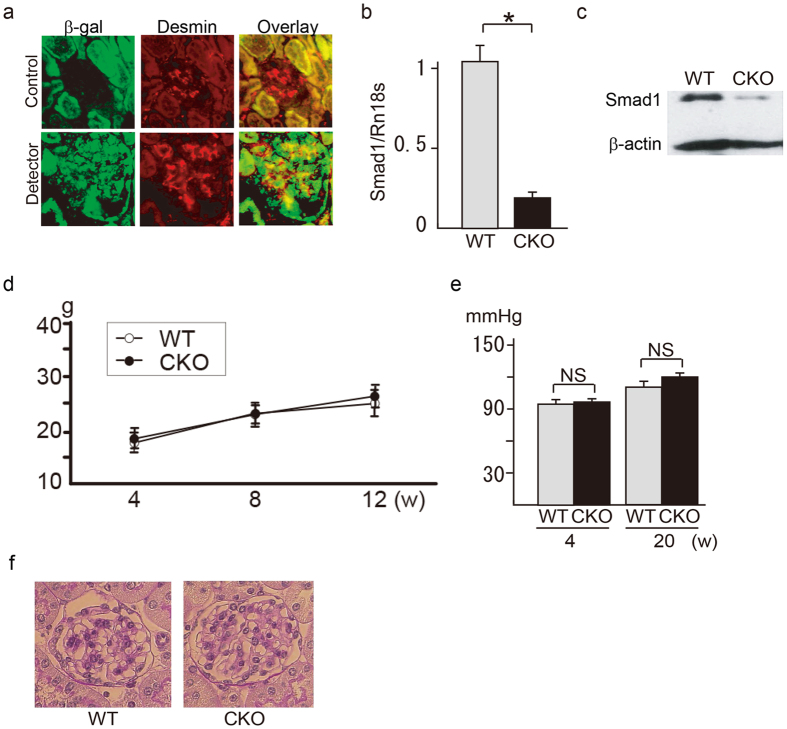
Generation and characterization of *Smad1*-CKO mice. (**a**) Tamoxifen-inducible LacZ expression of glomeruli in *ROSA26R Cre* reporter/*R26CreERT2* mice. Mice were sacrificed after 1 week of tamoxifen treatment. (**b**) qPCR analysis of Smad1 in the glomeruli isolated from LCM. The values are expressed as the mean ± SD. (*P < 0.05 compared to WT mice; n = 3). (**c**) Western blot analysis of Smad1 in the glomeruli isolated using the magnetic beads perfusion method. Body weight (**d**) and blood pressure (**e**) in control mice and CKO mice at the indicated times. (n = 4–6) (**f**) Histological analysis of the glomeruli of mice at 10 weeks. Representative sections from each mice kidney are shown (n = 4–6).

We initially crossed *Rosa26*^*CreERT2*^ mice with *Smad1*^*flox/−*^ mice, as conventional deletion of the *Smad1* gene results in early embryonic lethality prior to E10.5 and before kidney organogenesis. First, we confirmed Cre expression in the kidneys of *Rosa26-CreERT2* transgenic mice using lacZ detection. Adult *R26R/R26-CreERT2* mice were given tamoxifen for 5 consecutive days, and recombination of the lacZ reporter was analysed 7 days after the last tamoxifen administration. In contrast to wild type (WT) mice, tamoxifen-administered *Rosa26*^*CreERT2*^ mice showed lacZ expression in the glomeruli. Double immunostaining for desmin and β-galactosidase indicated that tamoxifen also induced transgene expression in MCs (Fig. 3a). Next, we exploited a tamoxifen-inducible knockout system by crossing *Rosa26*^*CreERT2/+*^ mice with *Smad1*^*flox/−*^mice to generate *Rosa26*^*CreERT2/+*^, *Smad1*^*flox/−*^ (*Smad1*-CKO) mice. We assessed Smad1 expression in the glomeruli of *Smad1*-CKO mice. QPCR and western blot analysis showed significantly decreased Smad1 expression in the glomeruli of *Smad1*-CKO mice (Fig. 3b,c). The conventional (i.e. non-conditional) *Smad1* knockout mice exhibit embryonic lethality; however, *Smad1*^*flox/−*^ or *Smad1*-CKO mice did not show any observable differences from WT mice. In addition, there were no differences in body weight or blood pressure between *Smad1*-CKO mice and WT mice (Fig. 3d,e). Moreover, there were no significant morphological changes detected in the kidneys (Fig. 3f). Thus, conditional *Smad1* gene ablation alone did not cause any phenotypic changes in the mice.

### *Smad1*-CKO mice with NTN exhibited reduced glomerular injury and reduced Smad1 activation

**Figure 4:**
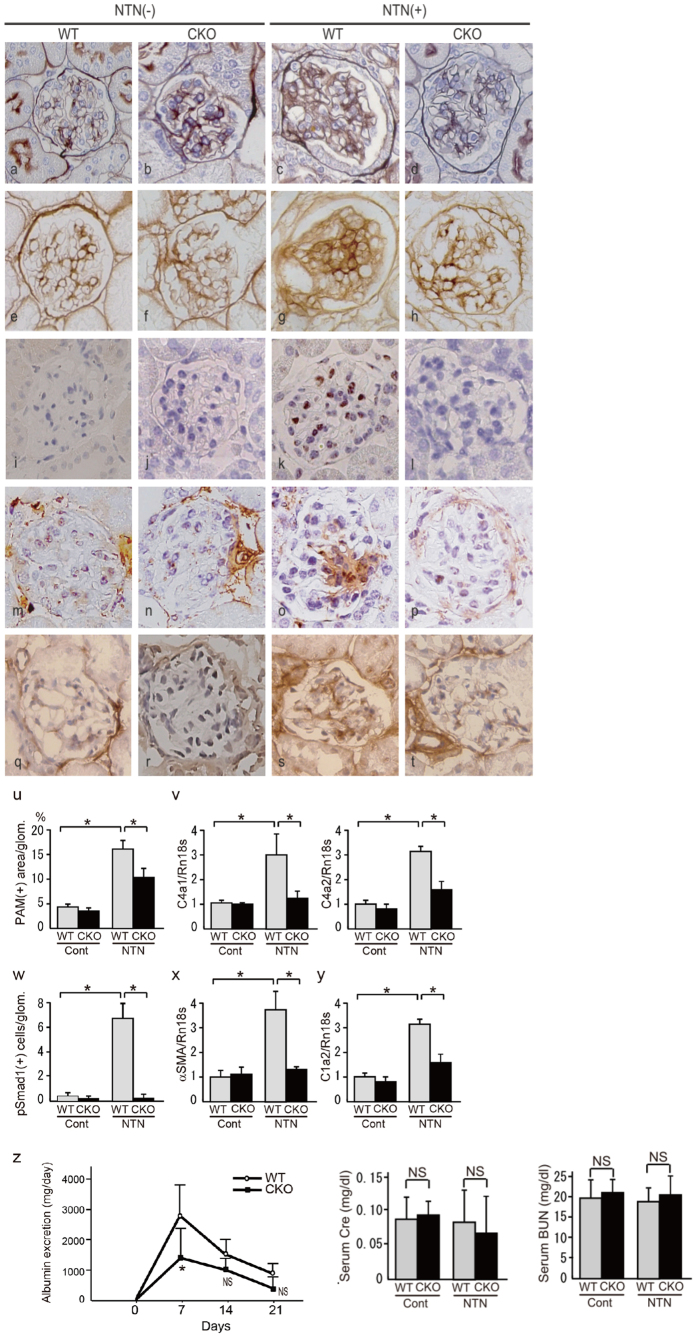
*Smad1*-CKO mice showed less renal injury and albuminuria than did wild-type mice with NTN. *Smad1*-CKO and WT mice treated with NTS-injection and control mice were dissected 21 weeks after treatment (n = 6 in each group). Light microscopic and immunohistochemical analyses were performed. Representative microscopic appearance of the glomerulus is shown. (**a**–**d**) PAM. (**e**–**h**) Col 4. (**i**–**l**) pSmad1. (**m**–**p**) Col1. (**q**–**t**) αSMA. Original magnification for all panels was ×400. (**u**) PAM staining-positive area of the glomeruli. We collected at least 20 glomeruli in each sample. (**v**) The expression levels of Col4 (Col4a1 and Col4a2) in the glomeruli 21 d after the initial NTS injection were analysed by qPCR and normalized to the expression of Rn18s. The values are expressed as the mean ± SD. *P < 0.05. (**w**) The number of cells positive for pSmad1. Fifty glomeruli were analysed for each sample. The values are expressed as the mean ± SD. *P < 0.05. The expression levels of αSMA (**x**) and Col1a2 (**y**) in the glomeruli 21 d after the initial NTS injection were analysed by qPCR and normalized to the expression of Rn18s. The values are expressed as the mean ± SD. *P < 0.05. (**z**) Time course of changes in urinary albumin excretion in the two groups – WT mice (○) and *Smad1*-CKO mice (●) – after injection of NTS, and serum Cre and BUN levels at day 21.. Statistical analysis for comparison between CKO and control mice was performed using unpaired two-tailed Student’s *t*-test. NS, not significant, *P < 0.05 (n = 4–5 mice/group).

We investigated the role of Smad1 in an experimental nephropathy model using *Smad1*-CKO mice. Twenty-one days after NTN-induction, we observed more extensive glomerulosclerosis in the NTN-induced WT mice (Fig. 4c), than in WT mice (Fig. 4a). We also noticed intense staining of Col4 in the WT mice after NTS treatment (Fig. 4g), which is consistent with pSmad1 staining (Fig. 4k,w). In *Smad1*-CKO mice, glomerulosclerosis and intense Col4 staining were markedly reduced after NTS treatment (Fig. 4d,h,u). Moreover, to determine whether distinct signalling cascades are involved in mesangial proliferation and sclerosis, we examined the molecules downstream of Smad1, αSMA and Col1^4^, and found an increase in Col1 and αSMA expression in NTS-treated mice (Fig. 4o,s), compared with that in non-treated WT mice (Fig. 4m,q). This increase was attenuated in *Smad1*-CKO mice (Fig. 4p,t). We then isolated glomeruli using LCM techniques, extracted mRNA, and analysed gene expression in the glomeruli. QPCR showed that Col4 expression was reduced in the *Smad1*-CKO mice compared with WT mice after NTS treatment (Fig. 4v). Similarly, increased glomerular αSMA and Col1 mRNA expressions in the NTN mice were almost eliminated in the *Smad1*-CKO mice (Fig. 4x,y). Next, we examined urinary albumin excretion (UAE) every 7 days and found that UAE in *Smad1*-CKO mice resulted in an albuminuria-lowering effect compared with that in WT mice; a significant decrease was observed at day 7 (Fig. 4z).

### *Smad1*-CKO mice did not show significant improvement in PDGF signalling and cell proliferation in NTN

**Figure 5:**
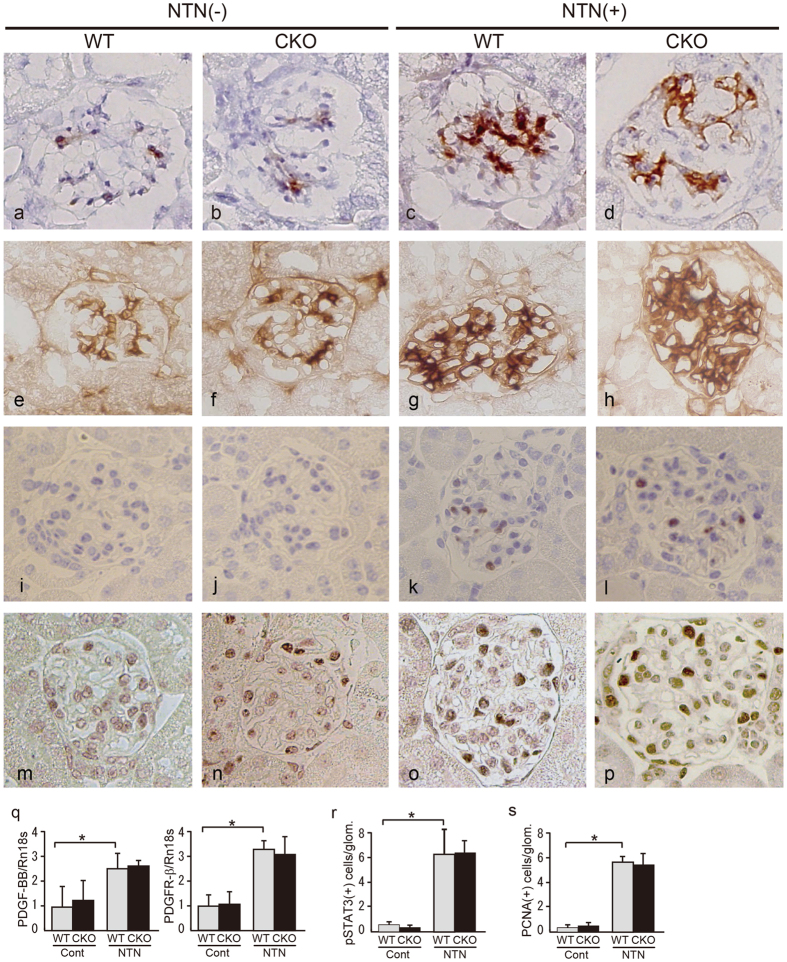
Proliferative changes were not observed in *Smad1*-CKO mice with NTN. *Smad1*-CKO and WT mice treated with NTS-injection and control mice were dissected 21 weeks after treatment (n = 6 in each group). Immunohistochemical analyses were performed. Representative microscopic appearance of the glomerulus is shown. (**a**–**d**) PDGF-B. (**e**–**h**) PDGFβR. (**i**–**l**) pSTAT3. (**m**–**p**) PCNA. Original magnification for all panels was ×400. We collected at least 20 glomeruli in each sample. (**q**) The expression levels of PDGF signals in the glomeruli 21 d after the initial NTS injection were analysed by qPCR and normalized to the expression of Rn18s. The values are expressed as the mean ± SD. *P < 0.05 The number of cells positive for pSTAT3 (**r**) and PCNA (**s**) was analyzed using fifty glomeruli from each sample. The values are expressed as the mean ± SD. *P < 0.05.

In addition, we examined whether the genetic ablation of *Smad1* affects PDGF signalling in glomeruli. The immunohistochemical staining patterns of PDGF-BB and PDGFβR (Fig. 5a,b,e,f) were not different between the WT and *Smad1*-CKO mice, and existence of Smad1 did not affect the expression levels of these signalling molecules after NTN treatment (Fig. 5c,d,g,h). QPCR of PDGF-BB showed similar results (Fig. 5q). We also found no difference in phosphorylated STAT3 (pSTAT3) expression or glomerular pSTAT3-positive cell number between the WT and *Smad1*-CKO mice with or without NTS treatment (Fig. 5i–l,r). Similarly, glomerular PCNA expression and PCNA-positive cell number were not significantly different between the WT and *Smad1*-CKO mice (Fig. 5m–p,s).

### Id2-VEGF signalling pathway is involved in cell proliferation independently of Smad1 in NTN

**Figure 6:**
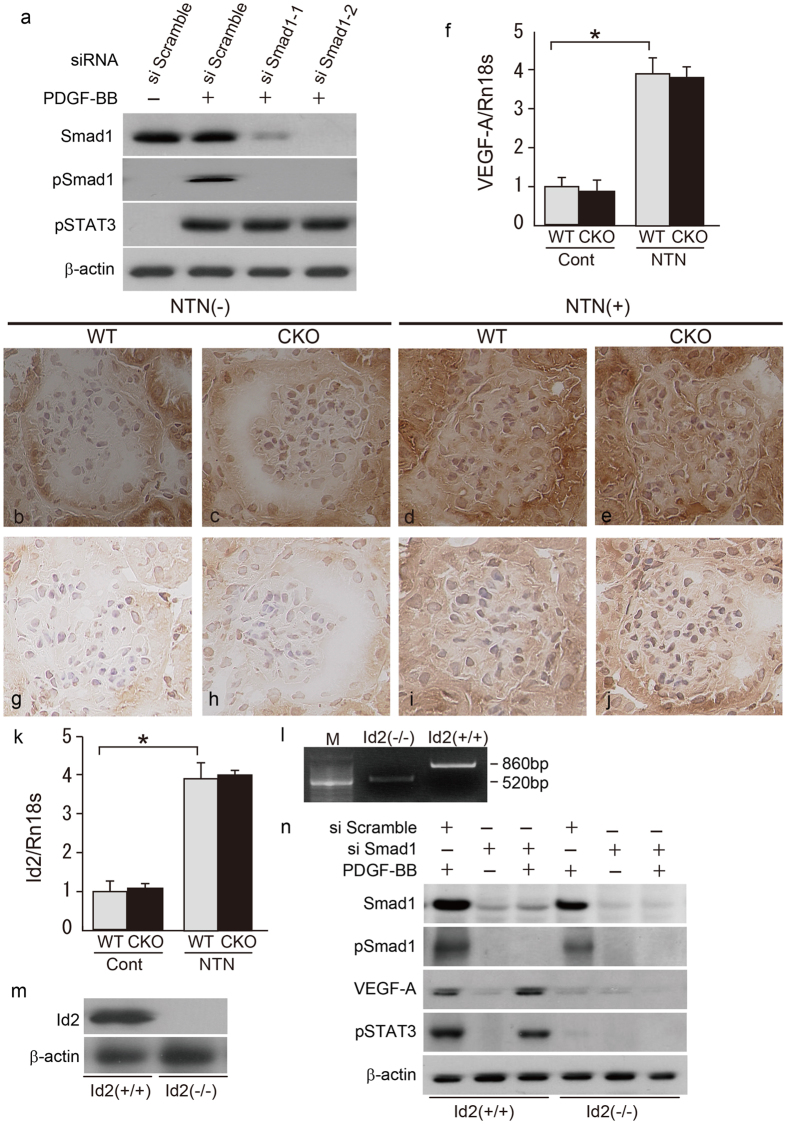
Evaluation for changes of PDGF signalling and cell proliferation under the influence of Smad1 silencing. (**a**) Effects of RNAi-mediated silencing of Smad1 on cell proliferation of MCs stimulated with PDGF-BB. Phosphorylation of Smad1 protein and STAT3 protein in MCs were monitored by western blotting. β-actin was used as a loading control. One of three independent experiments is shown. Immunohistochemical and quantitative analysis using qPCR of VEGF-A (**b**–**e**,**f**) and Id2 (**g**–**j**,**k**) in glomeruli in NTN. Data from qPCR were normalized to the expression of Rn18s. The values are expressed as the mean ± SD. *P < 0.05. (**l**) Confirmation of wild-type and null genotypes with PCR analysis of the *Id2* deletion on genomic DNA obtained from the MCs. M: marker. (**m**) Western blot analysis of Id2 protein expression in MCs. β-actin was used as a loading control. (**n**) Phosphorylation of STAT3 protein and secretion of VEGF-A protein in wild-type and Id2-null MCs were monitored by western blotting. β-actin was used as a loading control. One of three independent experiments is shown.

Although conditional *Smad1* deletion suppressed the glomerular ECM expansion, proliferative changes in glomeruli were not inhibited. From these results, existence of yet another molecular mechanism that activates proliferative changes in the absence of Smad1 protein in glomeruli was predicted. In some cells, STAT3 is phosphorylated and activated by vascular endothelial growth factor (VEGF)-A and influences cell proliferation^14^,^15^. Thus, we hypothesized that secretion of VEGF-A may occur under the activated PDGF-BB signalling pathway independently of Smad1 expression in NTN. To test this possibility, we first examined the relationship between Smad1 and pSTAT3 expression in MCs treated with PDGF-BB. Under the stimulation of PDGF-BB, STAT3 was phosphorylated independently of Smad1 expression and activation (Fig. 6a). Glomerular VEGF-A expression was remarkably increased in NTN (Fig. 6b–d). However, conditional knockout of *Smad1* did not influence the expression level of VEGF-A (Fig. 6e,f). Furthermore, we focused on the role of inhibitor of differentiation 2 (Id2), because Id2 was reported to induce VEGF-A secretion^16^,^17^. As expected, glomerular expression level of Id2 and VEGF-A were altered in tandem (Fig. 6g–k). In this model, ABP5 treatment showed a slight negative trend (not significant) at the number of pSTAT3- and PNCA-positive cells. In addition, expression levels of Id2 and VEGF-A in glomeruli were not reduced (Fig. S1). These results suggest that Id2-VEGF-A-STAT3 signalling pathway might be influenced by other receptors for PDGF-BB or other signalling pathways under inflammation (Fig. S3). To address whether Id2 transduces PDGF-BB signals to VEGF-A secretion, we examined the function of Id2 in MCs. We established primary cell lines from *Id2* knockout mice and wild type mice, respectively^18^ (Fig. 6l,m). In wild-type MCs, PDGF-BB stimulation induced VEGF-A secretion and phosphorylation of STAT3. In contrast, these effects were almost eliminated in Id2-null MCs (Fig. 6n). Moreover, we investigated the effects of PDGF-BB on cell proliferation of MCs via Id2. PDGF-BB time-dependently increased the cell number and DNA synthesis in wild-type MCs. In contrast, these proliferative changes were suppressed in Id2-null MCs (Fig. S2). Collectively, these results suggest that Id2 transduces the PDGF signal to VEGF-A and STAT3 independently of Smad1 expression in MCs.

## Discussion

Glomerulosclerosis is a common final lesion in various glomerular diseases and is characterized by mesangial ECM expansion. Overproduction of ECM is a critical feature in the progression of glomerular diseases toward end-stage renal disease, and halting the increase in ECM production may have some preventive effects for progression to renal insufficiency. As increasing evidence supports the role of glomerular cell proliferation in the development of glomerulosclerosis, control of proliferative changes in glomerular cells is also important. In many glomerular diseases, in particular GN, proliferation of intrinsic glomerular cells precedes glomerulosclerosis. In this study, we provided strong *in vivo* evidence that mesangial matrix expansion and the overexpression of ECM proteins such as α1 and α2 chains of Col4 were specifically inhibited by *Smad1* gene ablation, but that cell proliferation and STAT3 activation were not affected in an experimental model of progressive proliferative nephritis. Therefore, the signalling pathways responsible for mesangial matrix expansion and cell proliferation in glomeruli in accelerated nephrotoxic nephritis are not the same. This difference may hold the key to solving issues concerning plasticity in various kinds of GN.

During the progression of glomerular injuries, MCs can undergo phenotypic modulation, in which they markedly up-regulate the expression of Col4^19^. Col4 is a major component of the ECM, and we have demonstrated that Smad1 transcriptionally induces Col4 expression *in vitro*^4^. Thus we hypothesized that Smad1 may play a role in the progression towards glomerulosclerosis in GN. So far, no study has addressed the effect of gene ablation of *Smad1* in glomerular disease because conventional *Smad1* knockout mice are embryonic lethal. In addition, since there are no MC-specific Cre recombinases, we used *Rosa26*-*CreERT2* mice to generate *Smad1*-CKO mice. Furthermore, Cre recombinase is known to have possible toxic effects on hematopoietic cells. Thus, to avoid the influence of Cre toxicity, we first created an appropriate control group of mice that contained only activated Cre recombinase. We then injected the mice with NTS one month after the administration of tamoxifen (TM) when the complete recovery of haematological toxicity was demonstrated. Finally, we minimized the dose of TM^20^ administered. Some phenotypic abnormalities may be expected to appear in *Smad1*-CKO mice, as previous studies have shown that Smad1 is closely related to some cancers, bone formation and fibroblast-related skin diseases in adult mice^21^,^22^,^23^. However, these mice did not show abnormalities in any organs, including the glomeruli in the kidneys.

Although the Thy1 nephritis rat model is a good experimental model of rapidly progressive GN, this model often follows a self-limited course with spontaneous restitution of the glomerular architecture. Hence we selected the NTN model, which exhibits severe and progressive mesangial sclerosis, to investigate the key features that lead to irreversible and sustained changes in glomeruli. We sacrificed the mice 21 days after injection because glomerular injury was more prominent at day 21 than at day 7 or 14^11^. According to our previous study that found that Smad1 regulates the expression of Col4, Col1, and αSMA, resulting in ECM expansion *in vitro*^4^,^24^, we demonstrated a similar role of Smad1 in the NTN model. However, in contrast to the results observed in Thy1 nephritis, deletion of Smad1 had no effects on the proliferative changes in glomeruli in the NTN model, suggesting that activation of another signalling pathway may transduce PDGF-BB signals to the phosphorylation of STAT3 independently of Smad1 activation.

PDGF-BB is generally known as an essential mediator of mesangioproliferative nephritis. Overexpression of PDGF-BB increases the number of MCs and causes mesangial matrix expansion, leading to sclerosis of the glomeruli. STAT3 is recognized as a downstream target of PDGF-BB, and activated STAT3 contributes to cell-cycle proliferation, mitogenesis and chemotaxis^25^,^26^. However, it has remained undetermined what molecule directly transduces PDGF-BB signals to sclerotic and proliferative changes in progressive GN. In this study, we demonstrated clearly that Smad1 plays a critical role in the production of ECM proteins that subsequently lead to glomerulosclerosis. However, persistent activation of PDGF signalling may facilitate proliferative changes through the Id2-VEGF signalling pathway independently of Smad1 in MCs. Although APB5 is a highly workable monoclonal anti-PDGFβR antibody, PDGF-BB also binds other type-A receptors such as PDGFαR. Thus, there is other possibilities that Id2-VEGF signalling pathway is activated from other upstream molecules that is activated by PDGF or inflammatory stimulation (Fig. S3). Particularly in humans, glomerulonephritis can vary in severity. It can often be chronic – that is, it can last for a long time, and it can lead to irreversible damage in the glomeruli, interfering with kidney function and resulting in kidney failure. Therefore, it is critically important to elucidate the detailed mechanisms determining the irreversible glomerular changes related to poor prognoses. Further investigation regarding the role of the Id2-VEGF signalling pathway *in vivo* is required.

Effective resolution of inflammation requires cessation of proliferative changes in the process of GN. Some recent reports have heightened awareness of the fact that resolution is an active process that requires activation of endogenous programs that enable the host tissue to maintain homeostasis^26^,^27^,^28^. We here confirmed that Smad1 plays a pivotal role in ECM accumulation in glomerulonephritis using *Smad1*-CKO mice *in vivo*. Moreover, we also revealed that the Id2-VEGF-A-pSTAT3 pathway plays an important role in sustained cell proliferation of MCs independently of Smad1 expression. To provide a robust proof, NTN nephritis in conditional Id2-deleted mice should be investigated, because Id2–/– mice were born looked feeble, and their body size was very small compared with that of Id2+/+ or Id2+/– littermates^18^, and cannot bear the burden of administration of NTN. We think that this point is a limitation of this study. In addition, in the light of clinical assessment, the next task should involve longer-term observation of chronic proliferative glomerulonephritis in humans. Collectively, these results suggest that an imbalance between ECM accumulation and proliferative changes in the glomeruli may result in the irreversible progression of glomerulonephritis, leading to end stage kidney failure. Therapeutic approaches for irreversibly progressive glomerular diseases are currently limited to supportive therapy to slow the loss of function of the kidney. Our findings offer insights into the nature of the resolution of proliferative diseases and may provide potential therapeutic targets for inhibiting the progression of various renal diseases that lead to kidney failure. Combining with our overall findings summarized in Fig. S3, we can speculate that activation of Id2-VEGF signals under inflammation leading to sustained cell proliferation is a critical event in the development of glomerulosclerosis.

## Materials and Methods

### Animals

C57BL/6J mice were obtained from Shimizu Laboratory Animal Center (Hamamatsu, Japan). *Rosa26*^*CreERT2*^ mice were purchased from ARTEMIS Pharmaceuticals (Cologne, Germany)^29^. *Smad1*^*flox/−*^ mice^30^ were gifts from Dr. Anita B. Roberts and *R26R Cre* reporter mice (*R26R* mice)^31^ were gifts from Dr. P. Soriano. Mice were genotyped by PCR and were backcrossed for more than ten generations into C57BL/6 mice. All mice were housed under specific pathogen-free conditions. Mice were euthanized by carbon dioxide inhalation. All animal experiments were performed in accordance with institutional guidelines, and full details of the animal experimental protocols were approved and ethical permission was granted by the Review Board of Kyoto University.

### Induction of Accelerated NTN

From a previous paper^32^, decline of renal function was reported to depend on the amount of administrated NTS. Specifically, C57BL/6J mice with 0.2 ml of NTS daily injection were all dead within 10 days due to renal failure. Hence, we decided the dose of NTS as 0.1 ml. NTS^33^ was kindly provided by Dr. David J. Salant. Male mice (8 weeks old) were sensitized by subcutaneous injection of 1 mg of normal sheep IgG in Freund’s complete adjuvant in divided doses. Five days later, mice were injected with 0.1 ml of NTS daily for 3 days. Twenty-one days after the initial NTS injection, the mice were sacrificed, and their kidneys were examined.

### Treatment with Anti-PDGFβ-R Antibody in Accelerated NTN

A rat monoclonal anti-PDGFβR antibody (APB5) and its antagonistic effects on the PDGFβR signal transduction pathway *in vivo* and *in vitro* have been described previously^12^,^13^. Mice were injected intraperitoneally with 1 mg of APB5 or irrelevant isotype-matched control rat IgG once a day after the administration of nephrotoxic serum (NTS).

### Generation of conditional *Smad1* KO mice

To generate conditional inducible *Smad1* knockout mice, we bred *Smad1*^*flox/−*^ mice with *Rosa26*^*CreERT2*^ mice, which express a tamoxifen-inducible form of the Cre recombinase from the ubiquitous *Rosa26* locus. Tamoxifen (Sigma-Aldrich, MO) was dissolved in a sunflower oil/ethanol (9/1) mixture and injected intraperitoneally at a dose of 2 mg/20 g body weight for 5 consecutive days. Knockdown of Smad1 was confirmed using qPCR and Western blot.

### Histological Studies

One-third of each kidney was fixed in methyl Carnoy’s solution and embedded in paraffin. Sections (1 μm) were stained with periodic acid-Schiff and periodic acid methenamine silver (PAM) for routine histological examination. Portions of the kidney slices were mounted in Tissue-Tek OCT compound (Sakura Finetechnical, Tokyo, Japan) and snap-frozen. The other slices were fixed in 4% paraformaldehyde (PFA) and embedded in paraffin. Four micrometer-thick cryostat sections and 1-μm-thick paraffin sections were prepared. Paraffin embedded kidney sections were rehydrated, boiled for 15 min at 105 °C in citrate buffer (pH 6.0) and treated with 0.3% H_2_O_2_ in methanol for 30 min. Sections were blocked with the appropriate preimmune serum and then incubated with Avidin D and Biotin blocking solutions (Vector, CA). Sections were incubated first with the rabbit anti-PCNA, anti-PDGF-BB (Santa Cruz, CA), anti-VEGF-A (proteintech), and Id2 (Santa Cruz) polyclonal antibodies overnight at 4 °C, then with the appropriate biotinylated secondary antibodies and finally with the Vectastain Elite ABC kit (Vector). We used the ABC kit without boiling for the staining of the rabbit anti-α-SMA polyclonal antibody (Thermo Scientific, MA). Peroxidase conjugates were subsequently localized using diaminobenzidine. Instead of the ABC kit, the TSA system (Perkin-Elmer, Life Science, Ontario, Canada) was applied for the detection of the rabbit anti-pSmad1/5/8 (Millipore, CA) and anti-pSTAT3 antibodies (Cell Signaling Technology, MA). For detection of the goat anti-Col4 (PROGEN, Heidelberg, Germany) and rabbit anti-Col1 polyclonal antibodies (GeneTex, CA), the slides were treated with 0.1% protease VIII (Sigma-Aldrich) for 20 min at room temperature. Four-μm-thick cryostat kidney sections were fixed in cold acetone for 10 min and treated with 0.3% hydrogen peroxide in methanol for 30 min. After an appropriate blocking step, the sections were incubated with the rabbit anti-PDGF-B polyclonal antibody (Santa Cruz) overnight at 4 °C. The process for using the secondary antibodies, Vectastain Elite ABC kit and diaminobenzidine was the same as described above. When we used the rabbit anti-β-galactosidase (Millipore) and anti-Desmin polyclonal antibodies (Abcam, MA), we substituted cold acetone for 4% PFA at room temperature.

### Morphometric analyses

Staining density of mesangial matrix area as the PAM-positive area was analysed using Image-Pro Plus (Roper Industries, FL). The mesangial matrix fraction was determined as a percentage of the mesangial PAM-positive area per total glomerular surface area. For each animal, 50 glomeruli were analysed.

### LCM and qPCR analysis

Glomeruli were obtained from the mice by laser-manipulated microdissection methods using the Robot-Microbeam (P.A.L.M., Wolfratshausen, Germany) and an inverted microscope (CarlZeiss, Oberkochem, Germany). For each experiment, 20–40 glomeruli were collected, and total RNA was extracted from the glomeruli using the Pico Pure RNA Isolation Kit (Molecular Devices, CA) according to the manufacturer’s protocol. Reverse transcription from mRNA to cDNA was performed using SuperScript reverse transcription kits (Invitrogen, CA). Amplification was conducted in an ABI Prism 7900 Sequence Detection System (Roche, Basel, Switzerland) using TaqMan gene expression assays (Applied Biosystems, CA). Normalization was performed using Rn18s as internal standards. TaqMan assay ID number used for PCR amplification for each gene transcript were listed in table 1. For αSMA, custom TaqMan Gene Expression Assays were used that had the following primers and probes: forward primer 5′-ACCGACTACCTCATGAAGATCCT-3′, reverse primer 5′-GCACAGCTTCTCCTTGATGTCA-3′. The cycling parameters were 10 minutes at 95 °C, followed by 50 cycles of 15 sec at 95 °C and 60 sec at 60 °C. The other TaqMan gene expression assays used were as follows: Mm00484723_m1 (Smad1), Mm00483888_m1 (Col1 alpha 2 chain), Mm01210125_m1 (Col4 alpha 1 chain), Mm00802386_m1 (Col4 alpha 2 chain), Mm01298578_m1 (PDGF-B), Mm00435546_m1 (PDGFR-β), Mm00711781_m1 (Id2), and Mm00437306_m1 (VEGF-A).

### Western Blotting

Glomeruli were isolated using the magnetic beads perfusion method (Dynabead, Invitrogen/Dynal, Oslo, Norway)^34^. Isolated glomeruli were suspended in RIPA buffer (50 mM Tris, pH 7.5; 150 mM NaCl; 1% Nonidet P-40; 0.25% SDS; 1 mM Na_3_VO_4_; 2 mM EDTA; 1 mM phenylmethylsulfonyl fluoride; and 10 mg/ml of aprotinin) for 1 h at 4 °C. After centrifugation, the supernatants were used as total glomerular lysates. Each sample (10 μg) was subjected to SDS-PAGE. After electrophoresis, the proteins were transferred to nitrocellulose filters (Schleicher & Schuell). The blots were subsequently incubated with the rabbit anti-pSTAT3, Smad1 (Cell Signaling Technology), Id2 (Santa Cruz), VEGF-A (proteintech) and mouse β-actin (Cell Signaling Technology) polyclonal antibodies, followed by incubation with horseradish peroxidase-conjugated secondary antibody (Amersham Biosciences/GE Healthcare, Little Chalfont, UK). The immunoreactive bands were visualized using an enhanced chemiluminescent system (Amersham Biosciences). Quantitative analysis of the band density was performed using Image Gauge (Fuji Photo Film Co. Ltd., Kanagawa, Japan).

### Serum and Urine Examination

Urinary albumin excretion was measured at intervals from 0 to 21 days in 24-hour urine collection samples from mice housed in individual metabolic cages. During the urine collection, the mice were allowed free access to food and water. Albumin concentration in the urine was measured using the Albuwell kit (Exocell Inc., PA). Levels of serum creatinine and blood urea nitrogen were measured at day 21.

### Measurement of blood pressure

Systolic blood pressure (SBP) was measured every 2 weeks after surgery using a non-invasive computerized tail cuff system (Blood Pressure Analysis System BP- 98AW monitor, Softron Co., Ltd., Tokyo, Japan).

### Establishment of cell lines

Glomerular MCs were established from glomeruli isolated from *Id2* knockout^18^ mice (kindly provided by Dr. Y. Yokota) and their control littermates (strain background 129/sv) and were identified according to the method described previously^35^. MCs were maintained in B medium (a 3:1 mixture of minimal essential medium/F12 modified with trace elements) supplemented with 1 mM glutamine, penicillin at 100 units/ml, streptomycin at 100 mg/ml, and 20% fetal calf serum. The cultured cells fulfilled the generally accepted criteria for glomerular MCs^34^. Confirmation of wild-type and null genotypes were performed by using PCR with primers: Id2-S 5′-TCTGAGCTTATGTCGAATGATAAGC-3′, Id2-AS 5′-CGTGTTCTCCTGGTGAAATGGCTG-3′, and neo-1 5′-TCGTGCTTTACGGTATCGCCGCTC-3′.

### Small-interfering RNA

MCs (0.5 × 10^5^) were seeded into 12-well plates (Nunc) and were grown until they were 60% to 80% confluent. The small-interfering RNAs (siRNAs) for Smad1 (Dharmacon) or control scrambled siRNA (Dharmacon) were combined with INTERFERin transfection reagent (Polyplus), and the cells were transfected according to the recommended protocol with siRNA (20 nM final concentration). After 48 h of transfection, cells were starved in DMEM containing 0.5% BSA before treatment. After 24 h of addition of APB5 (100 ng/dl), the cells were stimulated with or without PDGF-BB (Calbiochem).

### BrdU proliferation assay

We performed BrdU proliferation assay to investigate whether Id2 affects the cell proliferation of MCs. MCs were were seeded into a 96-well microplate at a density of 10^4^ cells/well in B medium/10% FCS. The proliferation of MCs was determined at 24, 48, and 72 hours using a colorimetric immunoassay based on the measurement of BrdU incorporation during DNA synthesis, according to the instruction of CycLex BrdU Cellular ELISA Kit (MBL International).

### Statistical analysis

The data are expressed as the mean ± SD. Statistical analysis for comparison between CKO mice and control mice was performed using unpaired two-tailed Student’s *t*-test. Linear regression analysis was performed to evaluate the association between Smad1 and Col4 mRNA expression levels. A P-value <0.05 was considered to be statistically significant.

## Additional Information

**How to cite this article**: Araki, M. *et al.* Conditional Deletion of Smad1 Ameliorates Glomerular Injury in Progressive Glomerulonephritis. *Sci. Rep.* **6**, 31216; doi: 10.1038/srep31216 (2016).

## Supplementary Material

Supplementary Information

## References

[b1] Striker L. J., Doi T., Elliot S. & Striker G. E. The contribution of glomerular mesangial cells to progressive glomerulosclerosis. Semin Nephrol 9, 318–328 (1989). 2688008

[b2] Striker L. J., Peten E. P., Elliot S. J., Doi T. & Striker G. E. Mesangial cell turnover: effect of heparin and peptide growth factors. Lab Invest 64, 446–456 (1991). 2016850

[b3] Stokes M. B., Hudkins K. L., Zaharia V., Taneda S. & Alpers C. E. Up-regulation of extracellular matrix proteoglycans and collagen type I in human crescentic glomerulonephritis. Kidney Int 59, 532–542 (2001). 11168935 10.1046/j.1523-1755.2001.059002532.x

[b4] Abe H. *et al.* Type IV collagen is transcriptionally regulated by Smad1 under advanced glycation end product (AGE) stimulation. J Biol Chem 279, 14201–14206 (2004). 14732718 10.1074/jbc.M310427200

[b5] Matsuda M. *et al.* Gene expression of PDGF and PDGF receptor in various forms of glomerulonephritis. Am J Nephrol 17, 25–31 (1997). 9057949 10.1159/000169067

[b6] Langham R. G. *et al.* Over-expression of platelet-derived growth factor in human diabetic nephropathy. Nephrol Dial Transplant 18, 1392–1396 (2003). 12808179 10.1093/ndt/gfg177

[b7] Floege J. *et al.* Infusion of platelet-derived growth factor or basic fibroblast growth factor induces selective glomerular mesangial cell proliferation and matrix accumulation in rats. J Clin Invest 92, 2952–2962 (1993). 7902849 10.1172/JCI116918 PMC288499

[b8] Takahashi T. *et al.* Activation of STAT3/Smad1 is a key signaling pathway for progression to glomerulosclerosis in experimental glomerulonephritis. J Biol Chem 280, 7100–7106 (2005). 15591053 10.1074/jbc.M411064200

[b9] Zeisberg M. *et al.* BMP-7 counteracts TGF-beta1-induced epithelial-to-mesenchymal transition and reverses chronic renal injury. Nat Med 9, 964–968 (2003). 12808448 10.1038/nm888

[b10] Sumi E. *et al.* SRY-related HMG box 9 regulates the expression of Col4a2 through transactivating its enhancer element in mesangial cells. Am J Pathol 170, 1854–1864 (2007). 17525254 10.2353/ajpath.2007.060899 PMC1899455

[b11] Yanagita M. *et al.* Essential role of Gas6 for glomerular injury in nephrotoxic nephritis. J Clin Invest 110, 239–246 (2002). 12122116 10.1172/JCI14861 PMC151046

[b12] Sano H. *et al.* Study on PDGF receptor beta pathway in glomerular formation in neonate mice. Ann N Y Acad Sci 947, 303–305 (2001). 11795278 10.1111/j.1749-6632.2001.tb03951.x

[b13] Sano H. *et al.* Blockade of platelet-derived growth factor receptor-beta pathway induces apoptosis of vascular endothelial cells and disrupts glomerular capillary formation in neonatal mice. Am J Pathol 161, 135–143 (2002). 12107098 10.1016/s0002-9440(10)64165-x PMC1850709

[b14] Bartoli M. *et al.* VEGF differentially activates STAT3 in microvascular endothelial cells. FASEB J 17, 1562–1564 (2003). 12824281 10.1096/fj.02-1084fje

[b15] Wang H. *et al.* VEGF-mediated STAT3 activation inhibits retinal vascularization by down-regulating local erythropoietin expression. Am J Pathol 180, 1243–1253 (2012). 22230249 10.1016/j.ajpath.2011.11.031 PMC3349887

[b16] Lasorella A., Rothschild G., Yokota Y., Russell R. G. & Iavarone A. Id2 mediates tumor initiation, proliferation, and angiogenesis in Rb mutant mice. Mol Cell Biol 25, 3563–3574 (2005). 15831462 10.1128/MCB.25.9.3563-3574.2005 PMC1084294

[b17] Stallone G. *et al.* ID2-VEGF-related pathways in the pathogenesis of Kaposi’s sarcoma: a link disrupted by rapamycin. Am J Transplant 9, 558–566 (2009). 19260835 10.1111/j.1600-6143.2008.02537.x

[b18] Yokota Y. *et al.* Development of peripheral lymphoid organs and natural killer cells depends on the helix-loop-helix inhibitor Id2. Nature 397, 702–706 (1999). 10067894 10.1038/17812

[b19] Abe H., Iehara N., Utsunomiya K., Kita T. & Doi T. A vitamin D analog regulates mesangial cell smooth muscle phenotypes in a transforming growth factor-beta type II receptor-mediated manner. J Biol Chem 274, 20874–20878 (1999). 10409630 10.1074/jbc.274.30.20874

[b20] Higashi A. Y. *et al.* Direct hematological toxicity and illegitimate chromosomal recombination caused by the systemic activation of CreERT2. J Immunol 182, 5633–5640 (2009). 19380810 10.4049/jimmunol.0802413

[b21] Lechleider R. J. *et al.* Targeted mutagenesis of Smad1 reveals an essential role in chorioallantoic fusion. Dev Biol 240, 157–167 (2001). 11784053 10.1006/dbio.2001.0469

[b22] Huang S., Flanders K. C. & Roberts A. B. Characterization of the mouse Smad1 gene and its expression pattern in adult mouse tissues. Gene 258, 43–53 (2000). 11111041 10.1016/s0378-1119(00)00396-6

[b23] Vrljicak P. *et al.* Smad expression during kidney development. Am J Physiol Renal Physiol 286, F625–F633 (2004). 14656760 10.1152/ajprenal.00152.2003

[b24] Abe H. *et al.* Scleraxis modulates bone morphogenetic protein 4 (BMP4)-Smad1 protein-smooth muscle α-actin (SMA) signal transduction in diabetic nephropathy. J Biol Chem 287, 20430–20442 (2012). 22474292 10.1074/jbc.M111.275610 PMC3370223

[b25] Vignais M. L., Sadowski H. B., Watling D., Rogers N. C. & Gilman M. Platelet-derived growth factor induces phosphorylation of multiple JAK family kinases and STAT proteins. Mol Cell Biol 16, 1759–1769 (1996). 8657151 10.1128/mcb.16.4.1759 PMC231162

[b26] Andrae J., Gallini R. & Betsholtz C. Role of platelet-derived growth factors in physiology and medicine. Genes Dev 22, 1276–1312 (2008). 18483217 10.1101/gad.1653708 PMC2732412

[b27] Serhan C. N. A search for endogenous mechanisms of anti-inflammation uncovers novel chemical mediators: missing links to resolution. Histochem Cell Biol 122, 305–321 (2004). 15322859 10.1007/s00418-004-0695-8

[b28] Gilroy D. W., Lawrence T., Perretti M. & Rossi A. G. Inflammatory resolution: new opportunities for drug discovery. Nat Rev Drug Discov 3, 401–416 (2004). 15136788 10.1038/nrd1383

[b29] Seibler J. *et al.* Rapid generation of inducible mouse mutants. Nucleic Acids Res 31, e12 (2003). 12582257 10.1093/nar/gng012 PMC150244

[b30] Huang S. *et al.* Conditional knockout of the Smad1 gene. Genesis 32, 76–79 (2002). 11857782 10.1002/gene.10059

[b31] Soriano P. Generalized lacZ expression with the ROSA26 Cre reporter strain. Nat Genet 21, 70–71 (1999). 9916792 10.1038/5007

[b32] Chen S. M. *et al.* Induction of nephrotoxic serum nephritis in inbred mice and suppressive effect of colchicine on the development of this nephritis. Pharmacol Res 45, 319–324 (2002). 12030796 10.1006/phrs.2002.0948

[b33] Topham P. S. *et al.* Lack of chemokine receptor CCR1 enhances Th1 responses and glomerular injury during nephrotoxic nephritis. J Clin Invest 104, 1549–1557 (1999). 10587518 10.1172/JCI7707 PMC409862

[b34] Takemoto M. *et al.* A new method for large scale isolation of kidney glomeruli from mice. Am J Pathol 161, 799–805 (2002). 12213707 10.1016/S0002-9440(10)64239-3 PMC1867262

[b35] Davies M. The mesangial cell: a tissue culture view. Kidney Int 45, 320–327 (1994). 8164415 10.1038/ki.1994.41

