# Supplementary material for: Conditional Deletion of Smad1 Ameliorates Glomerular Injury in Progressive Glomerulonephritis
Source: Sci Rep. 2016 Aug 5;6:31216. doi: 10.1038/srep31216 (PMC4974558; doi:10.1038/srep31216)
Supplement: Supplementary Information [file srep31216-s1.pdf]

Conditional Deletion of Smad1 Ameliorates Glomerular Injury in Progressive Glomerulonephritis

Short running title: Postnatal deletion of Smad1-knockout ameliorates glomerular injury

Makoto Araki[1, 2], Takeshi Matsubara[1], Hideharu Abe[2*], Kazuo Torikoshi[1], Akira Mima[2], Noriyuki Iehara[1], Atsushi Fukatsu[1], Toru Kita[3], Hidenori Arai[4] and Toshio Doi[2]

[1]Department of Nephrology, Kyoto University, Kyoto, Japan; [2]Department of Nephrology, Institute of Biomedical Sciences, Tokushima University Graduate School, Tokushima, Japan; [3]Kobe City Medical Center General Hospital, Kobe, Japan; [4]National Center for Geriatrics and Gerontology, Obu, Japan.

Address reprint requests to: *Hideharu Abe, M.D.,Ph.D.
Department of Nephrology, Institute of Biomedical Sciences, Tokushima University Graduate School, Tokushima, 770-8503, Japan
Tel: 81-88-633-7184, Fax: 81-88-633-9245,
E-mail: abeabe@tokushima-u.ac.jp.

Figure S1

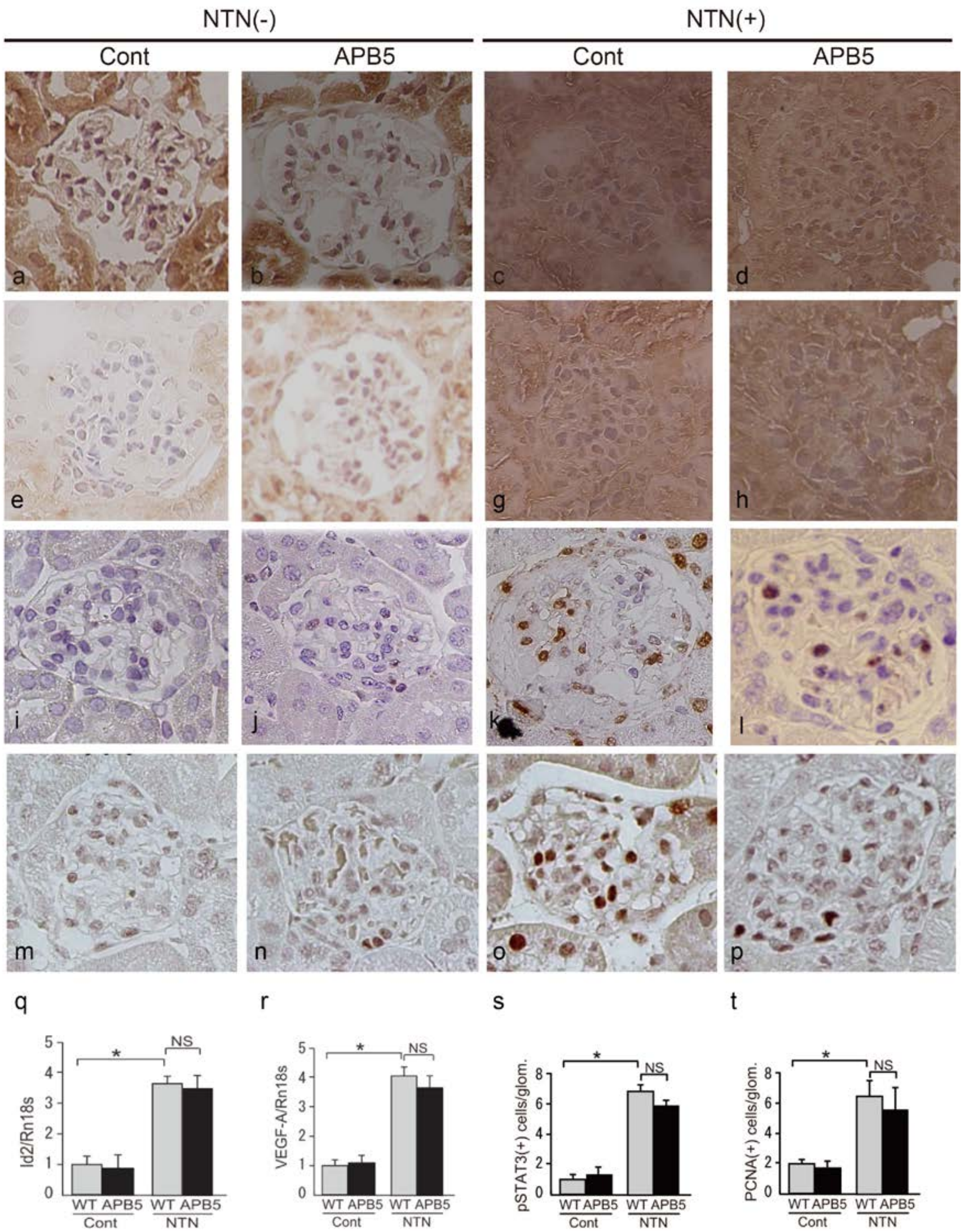

NTN(-)
NTN(+)
Cont
APB5
Cont
APB5
a
b
c
d
e
g
h
i
j
k
l
m
n
o
p
q
r
s
t
Id2/Rn18s
VEGF-A/Rn18s
pSTAT3(+) cells/glom.
PCNA(+) cells/glom.
*
NS
WT APB5 WT APB5
Cont NTN

Figure S2

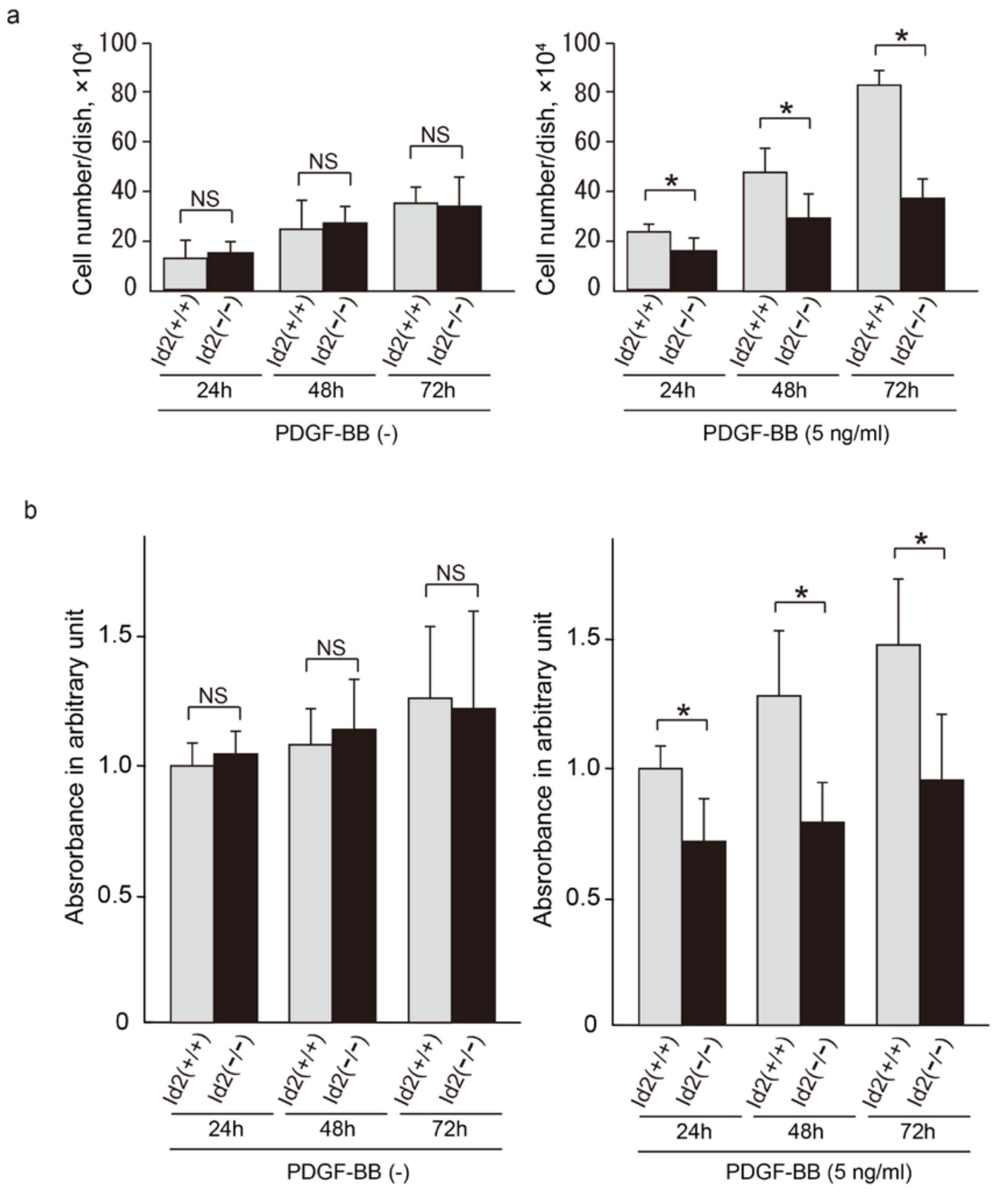
a
Cell number/dish, ×10⁴
100
80
60
40
20
0
NS
*
Id2(+/+)
Id2(−/−)
24h
48h
72h
PDGF-BB (-)
PDGF-BB (5 ng/ml)
b
Absrorbance in arbitrary unit
1.5
1.0
0.5
0
NS
*
Id2(+/+)
Id2(−/−)
24h
48h
72h
PDGF-BB (-)
PDGF-BB (5 ng/ml)

Figure S3

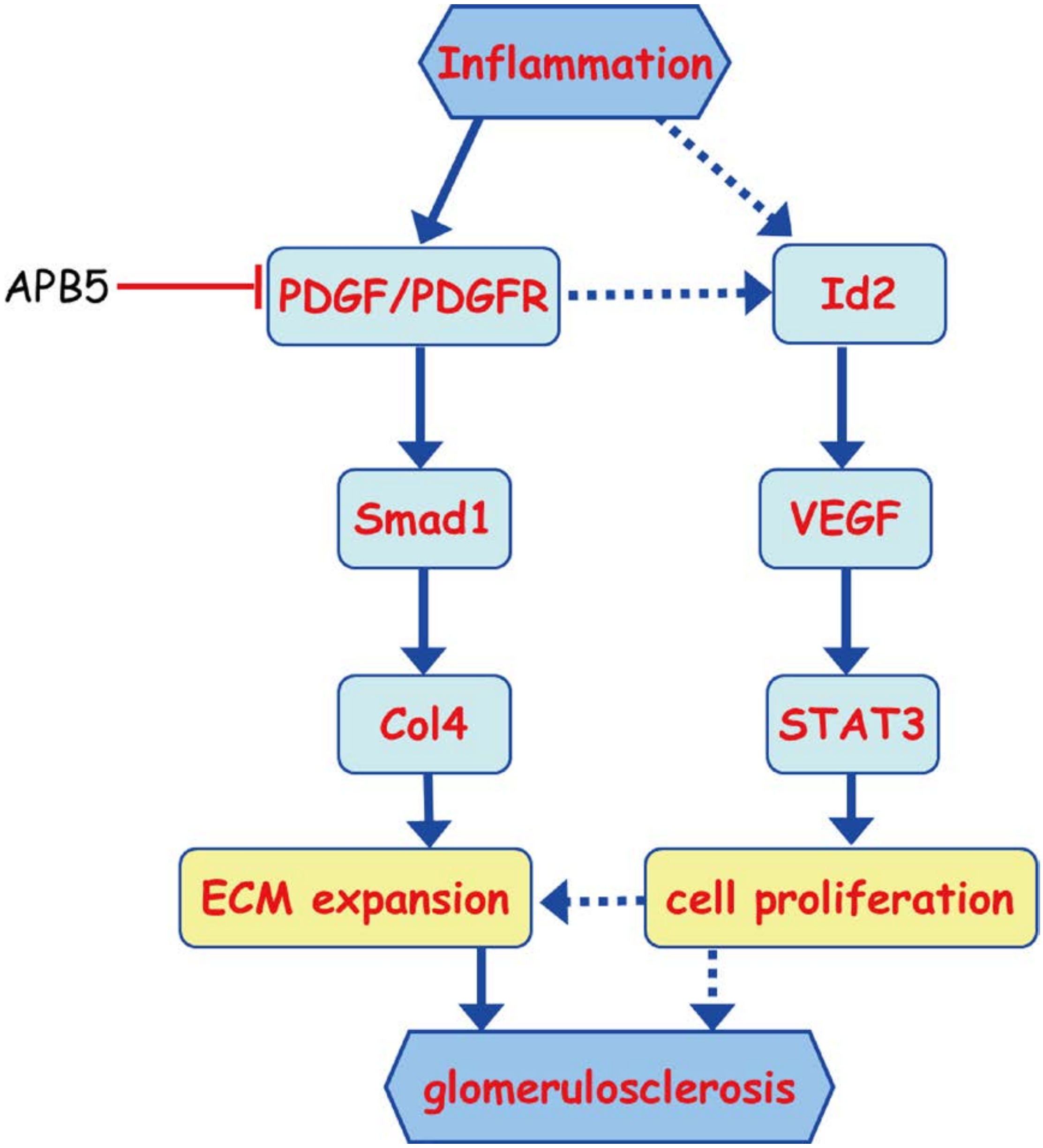

Figure Legends

Figure S1. Effects of PDGFβR inhibition on the proliferative changes in NTN. Representative immunohistochemical staining images were shown. (**a-d**) Id2. (**e-h**) VEGF-A. (**i-l**) pSTAT3. (**m-p**) PNCA. Quantitative analysis using qPCR of Id2 (**q**) and VEGF-A (**r**) in glomeruli in NTN. Data from qPCR were normalized to the expression of

Rn18s. The values are expressed as the mean ± SD. *P < 0.05. The number of cells positive for pSTAT3 (**s**) and PCNA (**t**) was analysed using fifty glomeruli from each sample. The values are expressed as the mean ± SD. *P < 0.05.

Figure S2. Effect of Id2 on the proliferation of MCs.
(**a**) Cell number was counted using a hemocytometer over a time course in the presence or absence of PDGF-BB in wild-type and Id2-null MCs. All assays were performed in triplicate. Quantitative data for cell numbers from five independent experiments are presented as mean ± SD. *P<0.05. (**b**) BrdU assay showing cell proliferation over a time course in the presence or absence of PDGF-BB in wild-type and Id2-null MCs. All assays were performed in triplicate and results represent as arbitrary units by optical absorption. The values are expressed as the mean ± SD of four experiments. *P < 0.05.

Figure S3. Proposed model for sustained cell proliferation under Id2-VEGF signalling pathway in glomerulonephritis.
PDGF and/or inflammation would potentially activate Id2-VEGF-A (broken arrows), resulting in sustained cell proliferation. Repeated or chronic activation of these signalling pathways may lead to the development of glomerulosclerosis in glomerulonephritis.
